# Nutritional Status of 8,128,014 Chilean and Immigrant Children and Adolescents Evaluated by the National Board of School Aid and Scholarships (JUNAEB) Between 2013 and 2023

**DOI:** 10.3390/nu17020327

**Published:** 2025-01-17

**Authors:** Edson Bustos-Arriagada, Fabián Vásquez, Karina Etchegaray-Armijo, Sandra López-Arana

**Affiliations:** Faculty of Medicine, Nutrition and Dietetics School, Universidad Finis Terrae, Pedro de Valdivia 1509, Providencia, Santiago 7501015, Chile; fvasquez@uft.cl (F.V.); ketchegaray@uft.cl (K.E.-A.); slopez@uft.cl (S.L.-A.)

**Keywords:** children, adolescents, immigrants, obesity, thinness, malnourished, stunting

## Abstract

Introduction: Nutritional issues, including overweight and obesity, along with the rising number of immigrants facing their own nutritional problems, continue to keep Chile on alert. Objective: To evaluate the epidemiological and nutritional status changes among Chilean and immigrant boys, girls, and adolescents (BGA) in schools evaluated by the National Board of School Aid and Scholarships (JUNAEB) from 2013 to 2023. Methods: This descriptive study analyzed individual, anonymous, and de-identified data on the nutritional status of BGA in pre-kindergarten, kindergarten, first grade, fifth grade, and the first year of high school using the JUNAEB Nutritional Map. Results: The sample consisted of 8,128,014 BGA, 49.2% women and 50.8% men. In 2013, immigrant BGA represented 0.4% of the total number of children evaluated, and by 2023, this percentage increased to 7.9%. It was observed that Chilean BGA had a lower proportion of thinness, risk of thinness, stunting, and normal weight, but a higher proportion of overnutrition compared to immigrants, similar to observations made during the COVID-19 pandemic. Comparisons by sex showed that Chilean and immigrant women had a lower percentage of thinness, risk of thinness, obesity, severe obesity, and stunting, and a higher proportion of normal weight when compared to men. Conclusions: The rise in immigrant BGA within the Chilean school system, together with the nutritional differences between both groups, highlights the need to consider these particularities when designing nutritional public policies in the health and education sectors.

## 1. Introduction

Chile has one of the highest prevalences of childhood overweight and obesity worldwide [1,2]. Additionally, immigration has increased substantially for more than a decade, becoming a concern at the state level in Chile.

Human mobility, driven by the search for better living conditions, employment, or the need to escape disasters and conflicts, has been a constant throughout history [3]. This situation leads to changes in the lifestyles of host countries, as well as adaptations by immigrants to their new environments. Migrant boys, girls, and adolescents (BGA) are a particularly vulnerable group with respect to nutritional and health problems [4,5,6].

By 2022, Chile had 1,625,074 immigrants, accounting for 8.8% of the country’s total population, which reflects a 3.9% increase from 2021. Among these, 210,521 are BGA under 19 years of age, representing 13.0% of the total estimated population [7]. According to UNICEF, BGA are migrating across Latin America and the Caribbean in record numbers. One in four people on the move in this region is a BGA, the highest proportion in the world [8]. These data reflect the significant presence of a young migrant population in Latin America, which has posed challenges in terms of educational integration, access to health care, and other essential services. Furthermore, these figures underscore the importance of developing public policies that address the specific needs of this growing population [7].

In Chile, the Ministry of Health (MINSAL), through the National Health Strategy 2011–2020 and the National Plan for the Prevention and Management of Childhood Obesity 2020–2030, has incorporated immigrant BGA into its initiatives. This inclusion is facilitated by the Consensual Intervention Plan (PIC), which establishes individualized and family-oriented goals to support BGA and their families in modifying eating behaviors, thereby improving their nutritional status [9].

We hypothesized that we would find a lower prevalence of obesity and a higher prevalence of thinness and stunting in immigrant BGA due to differences in their socioeconomic and cultural contexts [3]. The objective of this study was to evaluate the changes in epidemiological and nutritional status of Chilean and immigrant BGA evaluated by the National Board of School Aid and Scholarships (JUNAEB) between 2013 and 2023 in Chile.

## 2. Materials and Methods

This descriptive study independently examined the nutritional status of Chilean and immigrant BGA, as assessed by JUNAEB in its nutrition surveillance program, namely, “Mapa Nutricional”, between 2013 and 2023 (https://www.junaeb.cl/mapa-nutricional/, accessed on 9 January 2025). This analysis was conducted with authorization from the Subdepartment of Studies and Data Analysis—Planning Department—JUNAEB, and used individual, anonymous, and de-identified data in accordance with the Personal Data Protection Law of Chile (Law No. 19,628). The data are available in the JUNAEB Research Data Library [10,11], which aims to support research on topics of interest to this institution.

### 2.1. Database and Sampling

JUNAEB Nutritional Map between 2013 and 2023: nutritional status and sociodemographic characteristics. The available data include weight, height, Body Mass Index-for-age z-scores (zBMI/A), height-for-age z-scores (zH/A), nutritional status, height status, sex, nationality, type of educational establishment (public or subsidized private), geographic area (rural or urban), and region of the school of the BGA. These data are obtained from the educational levels of pre-kindergarten (around age 4), kindergarten (around age 5), 1st grade (around age 6), 5th grade (around age 10), and the 1st year of high school (around age 14) as reflected in the JUNAEB Nutritional Map for the mentioned years.

Since 1997, JUNAEB has collected nutritional information on students from public schools and subsidized private schools throughout Chile that receive financial support from the state. The exclusion criterion in our study was BGA without reported nationality (*n =* 14,917). The final sample included 8,128,014 nutritional assessments of BGA, 4,002,190 of whom were female and 4,125,824 were male.

### 2.2. Nutritional Status

The teachers were responsible for taking the anthropometric measurements. They received annual training in these measurements according to WHO standards, supervised by local experts [12]. According to the World Health Organization (WHO) 2006 and 2007 criteria [12], and the Ministry of Health of Chile [13], the BGA were classified based on their nutritional status as follows: thinness (zBMI/A ≤ −2), risk of thinness (zBMI/A ≤ −1), normal weight (zBMI/A > −1 and <1), overweight (zBMI/A ≥ 1), obesity (zBMI/A ≥ 2), and severe obesity (zBMI/A ≥ 3). Stunting was defined as zH/A ≤ −2. Additionally, the nutritional status was grouped into three categories: *Malnourished* (MN) -thinness and risk of thinness-, *normal weight* and *Overnutrition* (ON) -overweight, obesity and severe obesity-.

### 2.3. Sociodemographic Background

The educational level, nationality, type of school, geographic area, and region of the educational establishment were recorded by the teachers in charge of nutritional assessments according to the information provided to the educational establishment by the parents. Regarding nationality, only whether the students were Chilean or immigrants was reported, without specifying the country of origin in the case of immigrants.

### 2.4. Statistical Analysis

The percentages of BGA were reported by nationality and sex, and the prevalence of nutritional status indicators of thinness, risk of thinness, normal weight, overweight, obesity, and severe obesity was estimated, as well as stunting.

Categorical variables were described as percentages and analyzed using the two-sample proportion test. No variable adjustments were made. The level of statistical significance was set at *p* < 0.05. All statistical analyses were performed using Stata version 15 (Stata Corp., College Station, TX, USA).

### 2.5. Ethical Considerations

The protocol of this study was reviewed and approved with a waiver of informed consent by the Ethics Committee of the Universidad Finis Terrae (Protocol ID: 24-071; approval date: 16 December 2024; plenary session n°34).

## 3. Results

The total sample consisted of 8,128,014 nutritional assessments of BGA, 4,002,190 (49.2%) of whom were women and 4,125,824 (50.8%) were men, conducted between 2013 and 2023 on students attending pre-kindergarten, kindergarten, first grade, fifth grade, and the first year of high school in public and subsidized private schools throughout Chile. In 2013, immigrant BGA accounted for 0.4% (n = 2463) of the total number of those assessed, while in 2023, this percentage increased to 7.9% (n = 41,230) (Table 1).

**Figure 1:**
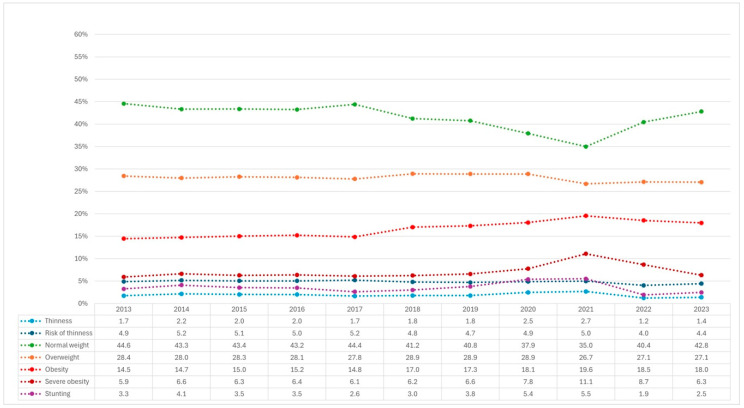
Nutritional status of Chilean boys, girls, and adolescents between 2013 and 2023, based on the JUNAEB.

The nutritional status of Chilean BGA (Figure 1) mainly shows a percentage increase in obesity and severe obesity, with rates of 14.5% and 5.9% in 2013 and 18.0% and 6.3% in 2023, respectively. Conversely, when comparing these same years, a decrease is observed in normal weight (44.6% vs. 42.8%), thinness (1.7% vs. 1.4%), risk of thinness (4.9% vs. 4.4%), and stunting (3.3% vs. 2.5%).

**Figure 2:**
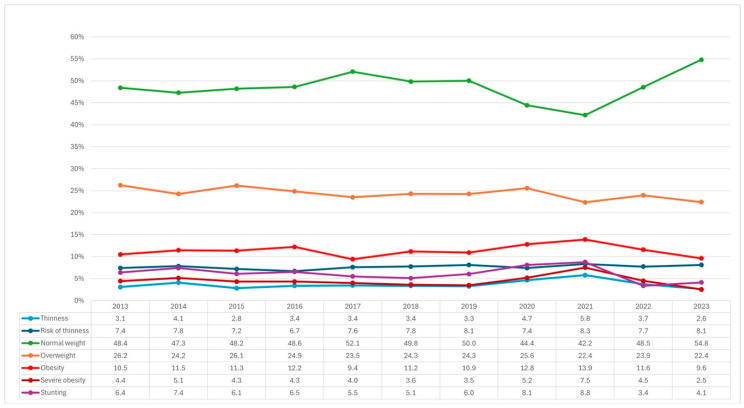
Nutritional status of immigrant boys, girls, and adolescents between 2013 and 2023, based on the JUNAEB.

The nutritional status of immigrant BGA (Figure 2), when comparing the years 2013 and 2023, shows a percentage increase in normal weight (48.4% vs. 54.8%) and a decrease in overweight (26.2% vs. 24.4%), obesity (10.5% vs. 9.6%), severe obesity (4.4% vs. 2.5%), and stunting (6.4% vs. 4.1%).

Additionally, it can be seen that during the years 2020 and 2021, which correspond to the COVID-19 pandemic period, when lockdowns and school closure measures were implemented in Chile, the nutritional status of both Chileans and immigrants underwent variations in their usual trajectories. Specifically, in Chilean and immigrant BGA, an increase in thinness, stunting, obesity, and severe obesity was observed, as well as a decrease in normal weight (Figure 1 and Figure 2).

**Figure 3:**
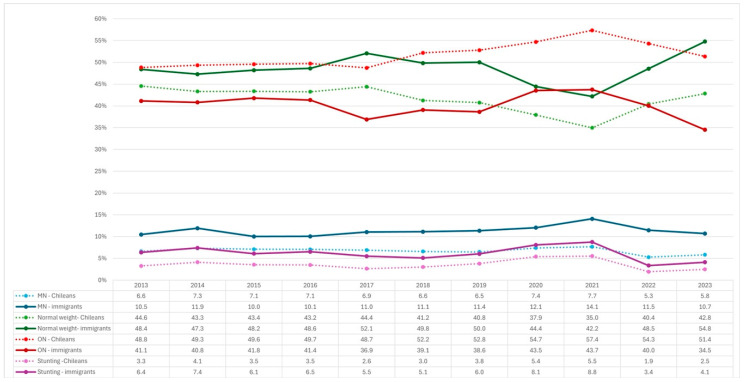
Grouped nutritional status of Chilean and immigrant boys, girls, and adolescents between 2013 and 2023, based on the JUNAEB.

When observing the grouped nutritional status of Chileans and immigrants (Figure 3), it is noteworthy that throughout the years studied, Chilean BGA exhibited a lower proportion of MN, stunting and normal weight, but a higher proportion of ON compared to immigrants.

When comparing nutritional status by sex within each group of Chilean and immigrant BGA (Table 2), it was generally observed that Chilean and immigrant females had a lower percentage of thinness, risk of thinness, obesity, severe obesity, and stunting and a higher proportion of normal weight compared to males. However, regarding the nutritional status of overweight, Chilean females had a higher percentage than Chilean males, which was contrary to what was observed in immigrant females when compared to their male peers. On the other hand, the significant differences observed were mainly among the Chilean BGA group, in which females presented a lower percentage of thinness (year 2021), risk of thinness (years 2014 to 2018), obesity (years 2013 to 2023), severe obesity (years 2013 to 2023), and stunting (year 2015), while they had a higher proportion of normal weight (years 2013 to 2023) and overweight (years 2015 to 2023) compared to Chilean males. In the immigrant BGA group, significant differences were observed in females, who had a lower percentage of obesity (years 2018, 2019, 2020, 2022, 2023) and severe obesity (years 2020 and 2021), as well as a higher proportion of normal weight (years 2013 to 2023) compared to males.

Additionally, comparisons by sex were made between the Chilean and immigrant groups (Supplementary Materials). It was observed that immigrant males and females had higher proportions of thinness, risk of thinness, normal weight, and stunting, but lower percentages of overweight, obesity, and severe obesity compared to Chilean males and females.

## 4. Discussion

Our study evaluated the epidemiological and nutritional status of BGA in Chile, including both the Chilean population and immigrants during the period 2013–2023, focusing on students attending schools that receive financial support from the Chilean State and are monitored by JUNAEB. The immigrant population in Chile was determined according to nationality of origin, regardless of the length of stay in the country. A significant increase in immigrant BGA was observed in the Chilean school system during this period, rising from 0.4% in 2013 to 7.9% in 2023. This increase in immigration and the years elapsed did not greatly modify the nutritional status trajectory of both groups of BGA, maintaining the trend in which immigrant BGA exhibited a lower proportion of ON but a higher proportion of normal weight, MN, and stunting compared to Chilean BGA. These results are especially relevant, considering that demographic changes and immigration, both in Chile and globally, are continuous and increasingly frequent phenomena, which requires countries to implement measures that address crucial issues such as food and nutrition, health, education, integration, and socioeconomic and cultural aspects, among others.

Several studies have suggested that immigrant BGA often face challenges in adapting to health and education systems, which can affect their nutrition, especially in terms of access to a balanced diet and primary health care [14,15], as well as present differences in their nutritional status when compared with BGA in the country to which they migrate [16,17,18].

In Chile, it has been reported that the immigrant population in 2022 comes mainly from countries such as Venezuela (32.8%), Peru (14.4%), Colombia (11.7%), Haiti (11.4%), Bolivia (9.1%), and Argentina (4.9%) [7]. These countries, despite having prevalences of ON (overweight, obesity, and severe obesity) in children that are lower than those in Chile [19,20], coexist with a greater double burden of malnutrition characterized by high prevalences of both deficit and excess [21,22]. This situation is reflected in the comparisons of our study between Chilean and immigrant BGA, where ON, despite being high among immigrants, does not reach the high percentages seen in the Chilean population, which does not mean that it is low in itself [20]. At the other extreme, while the MN and stunting are low in the Chilean population compared to those of the countries in the region, the same tendency can be seen when comparing the population of immigrants who come from these countries [20,22]. This situation has already been reported in other studies in which the migrant population from low- and middle-income countries facing social and cultural problems exhibit high prevalences of MN and stunting [4,23], as well as ON [24].

The analyses in our study included exceptional events such as the COVID-19 pandemic, which in Chile, as well as in much of the world, had significant repercussions on people’s health, mobility, and daily activities [25,26]. Additionally, increases in weight and BMI and a higher prevalence of obesity in children and adolescents were observed [27]. In this regard, the lockdowns and complete closure of schools in Chile during 2020 and 2021 modified the methodology for the nutritional assessment of BGA by JUNAEB. Thus, during the aforementioned years, anthropometric measurements were mainly carried out by the parents or caregivers of the BGA. This adaptation to the usual JUNAEB methodology is crucial for analyzing the data obtained, since variations in weight and height related to the body size of BGA are more sensitive to the results [28]. For this reason, a difference can be observed during this period in the usual trajectory of nutritional status for both Chilean and immigrant BGA, with higher rates of MN, ON, and stunting reported, as well as a lower prevalence of normal weight. This reported variation in nutritional status tends to be normalized from 2022 with the opening of schools.

Changes in the nutritional status of BGA during the pandemic period have been previously reported by other studies, which attribute ON mainly to modifications in eating habits, such as the intake of high-energy-density foods, ultra-processed foods, and foods rich in refined sugars, as well as to a reduction in physical activity [29,30,31]. Meanwhile, MN and stunting were associated with the suspension of school feeding programs, loss of family income sources, and food insecurity due to pandemic lockdowns [31,32,33].

There are notable differences in the nutritional status of males and females [24], as observed in our study. Females tend to exhibit a lower prevalence of thinness, risk of thinness, stunting [34], obesity, and severe obesity [24] than males. Studies consistently report disparities in childhood obesity prevalence between the sexes, with higher rates in males than in females when using the World Health Organization (WHO) 2007 growth standards, compared to the nutritional classification based on the tables of the Centers for Disease Control and Prevention (CDC) 2000 and the International Obesity Task Force (IOTF or Cole-IOTF) [35,36]. These findings suggest that international standards may be insufficient to address current populations effectively. Therefore, a unified gold standard should be developed, considering geographic region, ethnic diversity, and growth stages in children and adolescents [36].

Between 1990 and 2022, the most significant increases in childhood and adolescent obesity were reported in Polynesia, Micronesia, the Caribbean, Brunei, and Chile. By 2022, Chile and Qatar had the highest combined prevalence of thinness and obesity among school-aged male children and adolescents. In most countries, the rise in the double burden of malnutrition in BGA was primarily driven by an increase in obesity, while the decrease was attributed to reductions in underweight or thinness [37].

Our study assessed nutritional status over 11 years (2013–2023), and we found a significant increase in immigrant BGA in the Chilean school system, as well as differences in the trajectory of nutritional status between Chilean and immigrant BGA. Both groups exhibit high percentages of ON, but the percentages are even higher among Chilean BGA. On the contrary, MN, stunting, and normal weight are more prevalent in the immigrant population. The COVID-19 pandemic lockdown period in Chile during 2020 and 2021 led to changes in the nutritional status reports for both Chilean and immigrant BGA. This period saw an increase in the prevalences of ON, MN, and stunting. These issues normalized when the usual methodology of anthropometric assessments was resumed with the reopening of schools in 2022. Finally, found that when comparing each group of Chilean and immigrant BGA by sex, Chilean females in certain years exhibited a lower percentage of MN, stunting, obesity, and severe obesity, but a higher proportion of normal weight and overweight compared to Chilean males. Additionally, immigrant females in some years presented a lower percentage of obesity and severe obesity and a higher proportion of normal weight when compared to the group of male immigrants.

Among the main strengths of our study is the fact that it includes a nationally representative sample of BGA attending schools that receive financial support from the Chilean State and who have been under continuous nutritional monitoring since 1997 by the JUNAEB. This information has been relevant to different governments for the development of public policies in both education and health. However, there are also limitations, which mainly stem from the training of the personnel who carry out the anthropometric measurements. Future research should focus on carrying out continuous interventions at the school level in both the feeding and nutrition of BGA attending schools in Chile in order to reduce existing gaps and improve the nutritional status of all BGA.

## 5. Conclusions

There has been a considerable increase in immigrant BGA in the Chilean school system, accompanied by nutritional differences between both groups, which in the case of the immigrant population, is associated with a high double burden of malnutrition (MN and ON). In the Chilean population, nutritional problems mainly stem from ON, which is among the highest in the world. The information collected highlights the need to consider the particularities of both Chilean and immigrant BGA when designing nutritional public policies in the educational and health sectors, with the aim of strengthening and increasing physical activity and healthy eating programs in Chilean schools to combat malnutrition in all its forms.

Several successful interventions globally can serve as models for improving nutritional status among school-aged children. For instance, a school-based intervention analyzed in the Generation R Study cohort demonstrated long-term benefits in preventing childhood overweight by promoting healthy behaviors and creating supportive environments for students [38]. In Brazil, the National School Feeding Program (PNAE) mandates the inclusion of fresh, locally sourced foods in school meals, which has improved the dietary diversity of children and reduced malnutrition rates [39]. Similarly, in the United States, the implementation of the Healthy, Hunger-Free Kids Act (HHFKA) has enhanced the nutritional quality of school lunches, contributing to a reduction in childhood obesity prevalence among low-income families [40]. Adapting these strategies to the Chilean context—integrating culturally appropriate meals and targeted programs for immigrant children—could effectively address the double burden of malnutrition.

## Figures and Tables

**Table 1:** Nutritional assessments of Chilean and immigrant boys, girls, and adolescents between 2013 and 2023, based on the JUNAEB.

Years	Chileans n (%)	Immigrants n (%)	Total
2013	674,908 (99.6)	2463 (0.4)	677,371
2014	751,202 (99.5)	3940 (0.5)	755,142
2015	735,529 (99.1)	6745 (0.9)	742,274
2016	751,061 (98.7)	9527 (1.3)	760,588
2017	786,686 (98.4)	13,020 (1.6)	799,706
2018	897,290 (97.0)	27,405 (3.0)	924,695
2019	878,040 (95.4)	42,359 (4.6)	920,399
2020	699,661 (95.0)	36,700 (5.0)	736,361
2021	624,548 (95.1)	32,480 (4.9)	657,028
2022	587,387 (93.2)	43,164 (6.8)	630,551
2023	482,669 (92.1)	41,230 (7.9)	523,899
	7,868,981	259,033	8,128,014

**Table 2:** Nutritional status by sex of Chilean and immigrant boys, girls, and adolescents between 2013 and 2023, based on the JUNAEB.

Nutritional Status	Chileans			Immigrants		
	Male n (%)	Female n (%)	*p*	Male n (%)	Female n (%)	*p*
Thinness						
2013	6369 (1.9)	5461 (1.6)	0.216	49 (3.6)	27 (2.5)	0.794
2014	8676 (2.3)	7653 (2.1)	0.385	98 (4.6)	63 (3.5)	0.733
2015	8386 (2.2)	6810 (1.9)	0.196	105 (2.8)	86 (2.9)	0.967
2016	8400 (2.2)	6985 (1.9)	0.192	177 (3.5)	142 (3.2)	0.882
2017	7559 (1.9)	5929 (1.5)	0.076	240 (3.4)	208 (3.5)	0.953
2018	9242 (2.0)	7761 (1.7)	0.149	512 (3.5)	409 (3.2)	0.802
2019	9133 (2.0)	7768 (1.7)	0.150	759 (3.3)	617 (3.1)	0.834
2020	10,440 (2.8)	8655 (2.4)	0.084	987 (4.9)	719 (4.4)	0.629
2021	10,573 (3.1)	8044 (2.5)	0.014	1155 (6.4)	722 (5.0)	0.209
2022	4785 (1.5)	3476 (1.1)	0.117	676 (2.8)	387 (2.1)	0.485
2023	4385 (1.6)	3411 (1.3)	0.274	669 (2.8)	409 (2.4)	0.691
Risk of thinness						
2013	17,128 (5.1)	16,007 (4.7)	0.092	90 (6.5)	92 (8.5)	0.608
2014	20,741 (5.4)	18,469 (4.9)	0.025	182 (8.5)	126 (7.0)	0.631
2015	20,091 (5.3)	17,542 (4.8)	0.027	285 (7.7)	199 (6.6)	0.646
2016	20,594 (5.3)	17,920 (4.8)	0.025	368 (7.2)	270 (6.1)	0.583
2017	22,788 (5.6)	19,278 (4.9)	0.001	570 (8.1)	419 (7.0)	0.519
2018	23,911 (5.1)	21,244 (4.7)	0.049	1165 (7.9)	960 (7.6)	0.797
2019	23,302 (5.0)	21,427 (4.7)	0.140	1859 (8.2)	1571 (8.0)	0.830
2020	19,076 (5.1)	17,851 (4.9)	0.378	1476 (7.3)	1240 (7.5)	0.842
2021	17,510 (5.2)	16,317 (5.1)	0.677	1557 (8.7)	1139 (7.9)	0.458
2022	14,300 (4.5)	12,663 (4.1)	0.106	1845 (7.6)	1408 (7.5)	0.914
2023	13,330 (5.0)	11,438 (4.5)	0.065	2059 (8.5)	1279 (7.5)	0.303
Normal weight						
2013	141,784 (42.1)	160,130 (47.0)	0.001	618 (44.8)	574 (53.0)	0.004
2014	155,694 (40.8)	171,606 (45.9)	0.001	967 (45.1)	896 (49.9)	0.038
2015	154,630 (41.1)	167,511 (45.8)	0.001	1663 (44.6)	1588 (52.6)	0.001
2016	159,617 (41.3)	169,748 (45.3)	0.001	2339 (45.7)	2292 (52.0)	0.001
2017	174,138 (42.9)	181,930 (46.3)	0.001	3532 (50.3)	3248 (54.2)	0.001
2018	183,960 (39.1)	199,761 (44.0)	0.001	6982 (47.4)	6671 (52.7)	0.001
2019	179,797 (38.5)	199,176 (44.0)	0.001	10,841 (47.7)	10,347 (52.7)	0.001
2020	130,920 (35.0)	150,734 (41.7)	0.001	8336 (41.4)	7968 (48.2)	0.001
2021	109,806 (32.4)	122,391 (38.5)	0.001	7173 (40.0)	6528 (45.1)	0.001
2022	124,882 (39.0)	135,801 (43.8)	0.001	12,585 (51.6)	10,545 (56.2)	0.001
2023	111,207 (41.7)	118,058 (45.9)	0.001	12,717 (52.7)	9868 (57.8)	0.001
Overweight						
2013	95,384 (28.3)	97,120 (28.5)	0.330	381 (27.6)	265 (24.5)	0.378
2014	106,398 (27.9)	104,773 (28.0)	0.608	504 (23.5)	451 (25.1)	0.564
2015	105,441 (28.0)	104,280 (28.5)	0.011	1009 (27.1)	754 (25.0)	0.321
2016	106,498 (27.6)	106,967 (28.6)	0.001	1296 (25.3)	1071 (24.3)	0.575
2017	109,721 (27.0)	111,882 (28.5)	0.001	1657 (23.6)	1403 (23.4)	0.896
2018	132,088 (28.1)	134,019 (29.5)	0.001	3562 (24.2)	3093 (24.4)	0.849
2019	131,546 (28.1)	132,343 (29.2)	0.001	5556 (24.4)	4714 (24.0)	0.637
2020	105,878 (28.3)	105,590 (29.2)	0.001	5210 (25.9)	4173 (25.2)	0.440
2021	88,218 (26.0)	85,747 (27.0)	0.001	3918 (21.8)	3342 (23.1)	0.185
2022	83,215 (26.0)	85,836 (27.7)	0.001	5467 (22.4)	4313 (23.0)	0.481
2023	69,182 (25.9)	70,608 (27.5)	0.001	5344 (22.1)	3890 (22.8)	0.425
Obesity						
2013	51,336 (15.3)	46,576 (13.7)	0.001	170 (12.3)	88 (8.1)	0.304
2014	59,132 (15.5)	51,903 (13.9)	0.001	268 (12.5)	183 (10.2)	0.453
2015	59,145 (15.7)	51,992 (14.2)	0.001	454 (12.2)	311 (10.3)	0.417
2016	61,097 (15.8)	54,351 (14.5)	0.001	675 (13.2)	486 (11.0)	0.259
2017	62,454 (15.4)	55,484 (14.1)	0.001	696 (9.9)	526 (8.8)	0.514
2018	84,817 (18.0)	70,930 (15.6)	0.001	1848 (12.5)	1215 (9.6)	0.013
2019	86,205 (18.4)	70,400 (15.6)	0.001	2732 (12.0)	1887 (9.6)	0.010
2020	72,866 (19.5)	58,135 (16.1)	0.001	2899 (14.4)	1795 (10.9)	0.001
2021	69,378 (20.5)	57,285 (18.0)	0.001	2633 (14.6)	1878 (13.0)	0.126
2022	60,683 (18.9)	52,590 (17.0)	0.001	2797 (11.5)	1675 (8.9)	0.006
2023	48,739 (18.3)	42,004 (16.3)	0.001	2565 (10.6)	1393 (8.2)	0.015
Severe obesity						
2013	24,621 (7.3)	15,455 (4.5)	0.001	72 (5.2)	37 (3.4)	0.670
2014	30,720 (8.1)	19,377 (5.2)	0.001	124 (5.8)	78 (4.3)	0.640
2015	28,689 (7.2)	17,757 (4.9)	0.001	210 (5.6)	81 (2.7)	0.299
2016	29,983 (7.8)	18,428 (4.9)	0.001	266 (5.2)	145 (3.3)	0.376
2017	29,724 (7.3)	18,819 (4.8)	0.001	333 (4.7)	188 (3.1)	0.377
2018	36,142 (7.7)	20,820 (4.6)	0.001	673 (4.6)	315 (2.5)	0.113
2019	37,624 (8.1)	21,678 (4.8)	0.001	991 (4.4)	485 (2.5)	0.072
2020	35,398 (9.5)	20,818 (5.8)	0.001	1250 (6.2)	647 (3.9)	0.035
2021	43,703 (12.9)	28,056 (8.8)	0.001	1561 (8.7)	874 (6.0)	0.016
2022	32,626 (10.2)	19,694 (6.4)	0.001	1045 (4.3)	421 (2.3)	0.067
2023	20,019 (7.5)	11,518 (4.5)	0.001	790 (3.3)	247 (1.5)	0.139
Stunting						
2013	11,709 (3.5)	10,489 (3.1)	0.096	109 (7.9)	48 (4.4)	0.423
2014	16,446 (4.3)	14,736 (3.9)	0.075	152 (7.1)	139 (7.7)	0.845
2015	14,355 (3.8)	12,125 (3.3)	0.028	251 (6.7)	160 (5.3)	0.564
2016	14,300 (3.7)	12,454 (3.3)	0.076	365 (7.1)	256 (5.8)	0.519
2017	11,105 (2.7)	10,322 (2.6)	0.648	412 (5.9)	304 (5.1)	0.644
2018	14,012 (3.0)	14,366 (3.2)	0.332	730 (5.0)	663 (5.3)	0.800
2019	17,485 (3.7)	18,364 (4.1)	0.050	1348 (5.9)	1209 (6.2)	0.750
2020	21,179 (5.7)	19,588 (5.4)	0.186	1658 (8.2)	1308 (7.9)	0.765
2021	19,813 (5.8)	17,554 (5.5)	0.210	1636 (9.1)	1205 (8.3)	0.456
2022	6352 (2.0)	6425 (2.1)	0.690	890 (3.7)	554 (3.0)	0.477
2023	6804 (2.6)	6936 (2.7)	0.715	1041 (4.3)	657 (3.9)	0.687

Two-sample proportion test. *p* < 0.05.

## Data Availability

The original contributions presented in the study are included in the article/Supplementary Material, further inquiries can be directed to the corresponding author.

## Supplementary Materials

The following supporting information can be downloaded at: https://www.mdpi.com/article/10.3390/nu17020327/s1, Table S1: Comparison of nutritional status between Chilean and immigrant male and female between 2013 and 2023, based on the JUNAEB.
