# Supplementary material for: Nutritional Status of 8,128,014 Chilean and Immigrant Children and Adolescents Evaluated by the National Board of School Aid and Scholarships (JUNAEB) Between 2013 and 2023
Source: Nutrients. 2025 Jan 17;17(2):327. doi: 10.3390/nu17020327 (PMC11767989; doi:10.3390/nu17020327)
Supplement: Supplementary file 1 [file nutrients-17-00327-s001.zip › nutrients-3381930-supplementary.pdf]

Supplementary table S1. Comparison of nutritional status between Chilean and immigrant male and female between 2013 and 2023, based on the JUNAEB.

Nutritional Status	Male			Female		
	Chileans n (%)	Immigrants n (%)	*p*	Chileans n (%)	Immigrants n (%)	*p*
Thinness						
2013	6,369 (1.9)	49 (3.6)	0.387	5,461 (1.6)	27 (2.5)	0.710
2014	8,676 (2.3)	98 (4.6)	0.133	7,653 (2.1)	63 (3.5)	0.441
2015	8,386 (2.2)	105 (2.8)	0.678	6,810 (1.9)	86 (2.9)	0.501
2016	8,400 (2.2)	177 (3.5)	0.246	6,985 (1.9)	142 (3.2)	0.265
2017	7,559 (1.9)	240 (3.4)	0.098	5,929 (1.5)	208 (3.5)	0.023
2018	9,242 (2.0)	512 (3.5)	0.021	7,761 (1.7)	409 (3.2)	0.025
2019	9,133 (2.0)	759 (3.3)	0.016	7,768 (1.7)	617 (3.1)	0.012
2020	10,440 (2.8)	987 (4.9)	0.001	8,655 (2.4)	719 (4.4)	0.001
2021	10,573 (3.1)	1,155 (6.4)	0.001	8,044 (2.5)	722 (5.0)	0.001
2022	4,785 (1.5)	676 (2.8)	0.013	3,476 (1.1)	387 (2.1)	0.087
2023	4,385 (1.6)	669 (2.8)	0.028	3,411 (1.3)	409 (2.4)	0.075
Risk of thinness						
2013	17,128 (5.1)	90 (6.5)	0.547	16,007 (4.7)	92 (8.5)	0.087
2014	20,741 (5.4)	182 (8.5)	0.066	18,469 (4.9)	126 (7.0)	0.277
2015	20,091 (5.3)	285 (7.7)	0.073	17,542 (4.8)	199 (6.6)	0.239
2016	20,594 (5.3)	368 (7.2)	0.108	17,920 (4.8)	270 (6.1)	0.322
2017	22,788 (5.6)	570 (8.1)	0.001	19,278 (4.9)	419 (7.0)	0.050
2018	23,911 (5.1)	1,165 (7.9)	0.001	21,244 (4.7)	960 (7.6)	0.001
2019	23,302 (5.0)	1,859 (8.2)	0.001	21,427 (4.7)	1,571 (8.0)	0.001
2020	19,076 (5.1)	1,476 (7.3)	0.001	17,851 (4.9)	1,240 (7.5)	0.001
2021	17,510 (5.2)	1,557 (8.7)	0.001	16,317 (5.1)	1,139 (7.9)	0.001
2022	14,300 (4.5)	1,845 (7.6)	0.001	12,663 (4.1)	1,408 (7.5)	0.001
2023	13,330 (5.0)	2,059 (8.5)	0.001	11,438 (4.5)	1,279 (7.5)	0.001
Normal weight						
2013	141,784 (42.1)	618 (44.8)	0.175	160,130 (47.0)	574 (53.0)	0.001
2014	155,694 (40.8)	967 (45.1)	0.007	171,606 (45.9)	896 (49.9)	0.018
2015	154,630 (41.1)	1,663 (44.6)	0.004	167,511 (45.8)	1,588 (52.6)	0.001
2016	159,617 (41.3)	2,339 (45.7)	0.001	169,748 (45.3)	2,292 (52.0)	0.001
2017	174,138 (42.9)	3,532 (50.3)	0.001	181,930 (46.3)	3,248 (54.2)	0.001
2018	183,960 (39.1)	6,982 (47.4)	0.001	199,761 (44.0)	6,671 (52.7)	0.001
2019	179,797 (38.5)	10,841 (47.7)	0.001	199,176 (44.0)	10,347 (52.7)	0.001
2020	130,920 (35.0)	8,336 (41.4)	0.001	150,734 (41.7)	7,968 (48.2)	0.001
2021	109,806 (32.4)	7,173 (40.0)	0.001	122,391 (38.5)	6,528 (45.1)	0.001
2022	124,882 (39.0)	12,585 (51.6)	0.001	135,801 (43.8)	10,545 (56.2)	0.001

2023	111,207 (41.7)	12,717 (52.7)	0.001	118,058 (45.9)	9,868 (57.8)	0.001
Overweight						
2013	95,384 (28.3)	381 (27.6)	0.762	97,120 (28.5)	265 (24.5)	0.150
2014	106,398 (27.9)	504 (23.5)	0.028	104,773 (28.0)	451 (25.1)	0.171
2015	105,441 (28.0)	1,009 (27.1)	0.526	104,280 (28.5)	754 (25.0)	0.034
2016	106,498 (27.6)	1,296 (25.3)	0.066	106,967 (28.6)	1,071 (24.3)	0.001
2017	109,721 (27.0)	1,657 (23.6)	0.002	111,882 (28.5)	1,403 (23.4)	0.001
2018	132,088 (28.1)	3,562 (24.2)	0.001	134,019 (29.5)	3,093 (24.4)	0.001
2019	131,546 (28.1)	5,556 (24.4)	0.001	132,343 (29.2)	4,714 (24.0)	0.001
2020	105,878 (28.3)	5,210 (25.9)	0.001	105,590 (29.2)	4,173 (25.2)	0.001
2021	88,218 (26.0)	3,918 (21.8)	0.001	85,747 (27.0)	3,342 (23.1)	0.001
2022	83,215 (26.0)	5,467 (22.4)	0.001	85,836 (27.7)	4,313 (23.0)	0.001
2023	69,182 (25.9)	5,344 (22.1)	0.001	70,608 (27.5)	3,890 (22.8)	0.001
Obesity						
2013	51,336 (15.3)	170 (12.3)	0.278	46,576 (13.7)	88 (8.1)	0.127
2014	59,132 (15.5)	268 (12.5)	0.176	51,903 (13.9)	183 (10.2)	0.148
2015	59,145 (15.7)	454 (12.2)	0.041	51,992 (14.2)	311 (10.3)	0.049
2016	61,097 (15.8)	675 (13.2)	0.065	54,351 (14.5)	486 (11.0)	0.029
2017	62,454 (15.4)	696 (9.9)	0.001	55,484 (14.1)	526 (8.8)	0.001
2018	84,817 (18.0)	1,848 (12.5)	0.001	70,930 (15.6)	1,215 (9.6)	0.001
2019	86,205 (18.4)	2,732 (12.0)	0.001	70,400 (15.6)	1,887 (9.6)	0.001
2020	72,866 (19.5)	2,899 (14.4)	0.001	58,135 (16.1)	1,795 (10.9)	0.001
2021	69,378 (20.5)	2,633 (14.6)	0.001	57,285 (18.0)	1,878 (13.0)	0.001
2022	60,683 (18.9)	2,797 (11.5)	0.001	52,590 (17.0)	1,675 (8.9)	0.001
2023	48,739 (18.3)	2,565 (10.6)	0.001	42,004 (16.3)	1,393 (8.2)	0.001
Severe obesity						
2013	24,621 (7.3)	72 (5.2)	0.494	15,455 (4.5)	37 (3.4)	0.747
2014	30,720 (8.1)	124 (5.8)	0.349	19,377 (5.2)	78 (4.3)	0.721
2015	28,689 (7.2)	210 (5.6)	0.371	17,757 (4.9)	81 (2.7)	0.360
2016	29,983 (7.8)	266 (5.2)	0.115	18,428 (4.9)	145 (3.3)	0.373
2017	29,724 (7.3)	333 (4.7)	0.069	18,819 (4.8)	188 (3.1)	0.277
2018	36,142 (7.7)	673 (4.6)	0.003	20,820 (4.6)	315 (2.5)	0.076
2019	37,624 (8.1)	991 (4.4)	0.001	21,678 (4.8)	485 (2.5)	0.019
2020	35,398 (9.5)	1,250 (6.2)	0.001	20,818 (5.8)	647 (3.9)	0.013
2021	43,703 (12.9)	1,561 (8.7)	0.001	28,056 (8.8)	874 (6.0)	0.004
2022	32,626 (10.2)	1,045 (4.3)	0.001	19,694 (6.4)	421 (2.3)	0.001
2023	20,019 (7.5)	790 (3.3)	0.001	11,518 (4.5)	247 (1.5)	0.001
Stunting						
2013	11,709 (3.5)	109 (7.9)	0.013	10,489 (3.1)	48 (4.4)	0.604
2014	16,446 (4.3)	152 (7.1)	0.091	14,736 (3.9)	139 (7.7)	0.022
2015	14,355 (3.8)	251 (6.7)	0.018	12,125 (3.3)	160 (5.3)	0.161
2016	14,300 (3.7)	365 (7.1)	0.001	12,454 (3.3)	256 (5.8)	0.028

2017	11,105 (2.7)	412 (5.9)	0.001	10,322 (2.6)	304 (5.1)	0.008
2018	14,012 (3.0)	730 (5.0)	0.002	14,366 (3.2)	663 (5.3)	0.003
2019	17,485 (3.7)	1,348 (5.9)	0.001	18,364 (4.1)	1,209 (6.2)	0.001
2020	21,179 (5.7)	1,658 (8.2)	0.001	19,588 (5.4)	1,308 (7.9)	0.001
2021	19,813 (5.8)	1,636 (9.1)	0.001	17,554 (5.5)	1,205 (8.3)	0.001
2022	6,352 (2.0)	890 (3.7)	0.001	6,425 (2.1)	554 (3.0)	0.163
2023	6,804 (2.6)	1,041 (4.3)	0.002	6,936 (2.7)	657 (3.9)	0.075

Two-sample proportion test. $p < 0.05$.
